# Acute abdomen caused by ingested chicken wishbone: a case report

**DOI:** 10.1186/1757-1626-2-64

**Published:** 2009-01-19

**Authors:** Faton T Hoxha, Shemsedin I Hashani, Driton S Komoni, Lumturije H Gashi-Luci, Fisnik I Kurshumliu, Medita SH Hashimi, Avdyl S Krasniqi

**Affiliations:** 1 Department of Abdominal Surgery, University Clinical Centre of Kosova, Prishtina, Republic of Kosovo; 2 Institute of Pathology, University Clinical Centre of Kosova, Prishtina, Republic of Kosovo; 3 Department of Anesthesiology, University Clinical Centre of Kosova, Prishtina, Republic of Kosovo

## Abstract

**Introduction:**

An ingested foreign body often passes the gastrointestinal tract without any complications. Foreign bodies, such as dentures, fish bones, chicken bones, and toothpicks, have been known to cause perforation of the GI tract.

**Case presentation:**

We are presenting a case of a fifty-year-old male with acute abdomen; diffuse fibro purulent peritonitis, i.e. ileum perforation, caused by accidentally ingesting a chicken wishbone. He was treated surgically with ileum resection, and temporary ileostomy. After four months, intestinal continuity was established in the second operation.

**Conclusion:**

Intestinal perforation by a chicken bone is rare and affects the left colon or distal ileum. The lack of information of ingestion and detection of chicken bones preoperatively are of interest to be considered in the differential diagnosis of acute abdomen, which in this case was treated surgically.

## Introduction

Ingesting a foreign body is not an uncommon occurrence and most foreign objects pass uninterrupted through the gastro intestinal tract without any complications [1]. In a few cases a patient's occurrence of bowel perforation leads to acute abdomen requiring surgical treatment [1,2]. Operative discovery remains in most cases [1]. Foreign bodies, such as dentures, fish bones, chicken bones, and toothpicks, have been known to cause perforation of the GI tract [1].

## Case presentation

We are presenting a case with acute abdomen, i.e. ileum perforation, caused by accidentally ingesting a chicken wishbone.

A fifty-year-old male was presented in the surgical emergency clinic with abdominal pain, nausea, and vomiting. The symptoms had started two days earlier. He used tobacco, but denied having consumed alcohol. The patient's height was 170 cm and his weight was 120 kg. His vital signs were: blood pressure 120/80 mmHg; pulse rate 90 beats/min.; respiration rate 19/min.; body temperature 38.5°C. His past medical history consisted of surgery for a thrombophlebitis in his right arm one year earlier.

Upon physical examination, a distended and diffusely tender abdomen with right lower abdomen rebound was revealed. The patient's skin and mucosa were pale.

The emergent laboratory tests revealed as follow: white blood cells (WBC): 12.6 × 10^9^/liter; serum creatinine: 126 mol/L; blood urea nitrogen: 8.6 mmol/L; blood sugar: 11.6 mmol/L; Na: 141 mmol/L; K: 4.0 mmol/L; Ca++: 1.08 mmol/L; urinalysis: a lot of mucus, 4–6 Leucocytes; some epithelial cells. Plain abdominal radiography showed mechanical obstruction. Urgent abdominal ultrasound revealed mechanical obstruction, dilated small bowels and free liquid in the peritoneal cavity.

**Figure 1:**
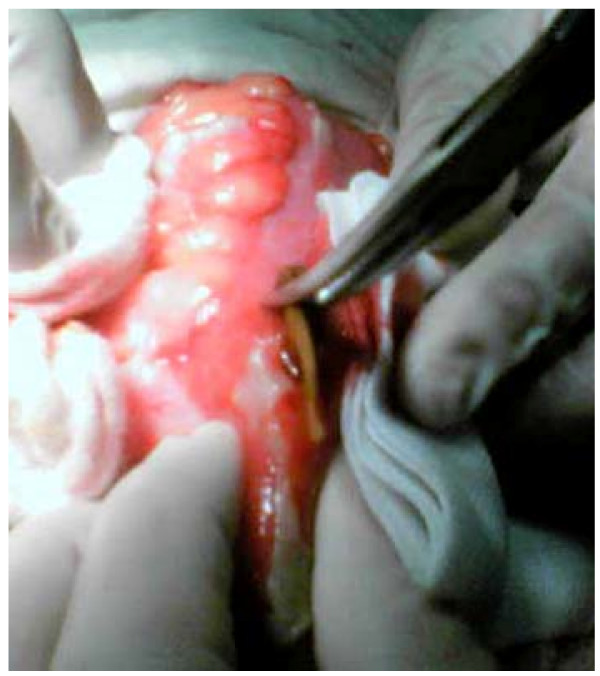
**Intra operative finding of perforated ileum with chicken wishbone**.

Laparotomy was performed in general anesthesia on the day the patient was admitted. Intra operative findings revealed diffuse fibro purulent peritonitis with adhesions between small bowels; and about 40 cm from Bauchini valve the presence of a sharp chicken wishbone perforated the ileum at the ante mesenteric site (Figure 1). The wall of that part of the ileum was thick and succulent. The patient was treated after the adhesiolisis with resection of the perforated distal ileum and ileum stoma. The postoperative treatment went well; the wound healed per secundum. *Clebsiela spp*. was isolated in the stained abdominal liquid and treated with the proper antibiotics. Four months after the first operation and losing 25 kg, the patient underwent reconstruction of bowel continuity, and discharged on the tenth postoperative day with normal bowel movements.

Retrospectively, after the first operation the patient admitted that four days earlier he had rapidly eaten and swallowed several mouthfuls of chicken meat without chewing and accidentally ingested a chicken bone.

## Discussion

Foreign bodies (FB) accidentally ingested mostly pass through the gastrointestinal tract (GT) without any consequences [3]. The most common objects are dentures, fish bones, chicken bones, toothpicks, and cocktail sticks. Very small percentages perforate the GI tract, which may occur from mouth to anus. A definitive preoperative history of foreign body ingestion is uncertain [1].

Small bowel perforations by FB are rarely diagnosed preoperatively because clinical symptoms are usually non-specific and mimic other surgical conditions, such as appendicitis and caecal diverticulitis [4]. Greater risk of perforation occurs at extreme ages, in those wearing dentures and orthodontic appliances [5], in patients with previous bowel pathology, or in alcoholic and psychiatric patients [6,7].

The risk of perforation is related to the length and the sharpness of the object [8].

Overeating, rapid eating, or a voracious appetite may be contributing factors for ingesting chicken bones. The mean time from ingestion to perforation was 10.4 days [5]. Most perforations occur at the narrowing and angulations of the GI tract [9]. The most common abdominal site of perforation is the distal ileum [1,4,10-12], caecum, and left colon [5,11,13,14], although an increased incidence of perforation has been reported in association with the Meckel diverticulum, the appendix, and/or mimicking diverticular disease [2,10,15-17].

The clinical presentation includes peritonitis, abdominal abscess formation [2], perineum and scrotal abscess [18], enterovesical fistulas, intestinal obstructions, and hemorrhage [2]. The most common preoperative diagnoses were acute abdomen of uncertain origin [5]. Our patient had a clinical presentation of acute abdomen with a suspicion of perforated appendicitis.

Patients with FB perforations in the stomach, duodenum, and large intestine were significantly more likely to be febrile, to have chronic symptoms, to have a normal total white blood cell count, and to be asymptomatic or present with an abdominal mass or abscess, compared to those with FB perforations in the jejunum and ileum [1].

The diagnosis was reached during laparotomy in more than 90% of the cases [1,5,11,12]. All cases had abdominal contamination and 66.7% had diffuse peritonitis [5].

Although the imaging of findings can be nonspecific, the identification of a chicken bone with an associated mass or extra luminal collection of gas in patients with clinical sign of peritonitis, mechanical bowel obstruction, or pneumoperitonem strongly suggests the diagnosis [9,13,19,20].

The treatment usually involves resection of the bowel, although occasionally repair has been described [9]. The most common treatment was simple suture of the defect [11]. The lack of conditions pre-disposing accidental ingestion of FB and no specific history of FB are of interest in these cases [12].

**Figure 2:**
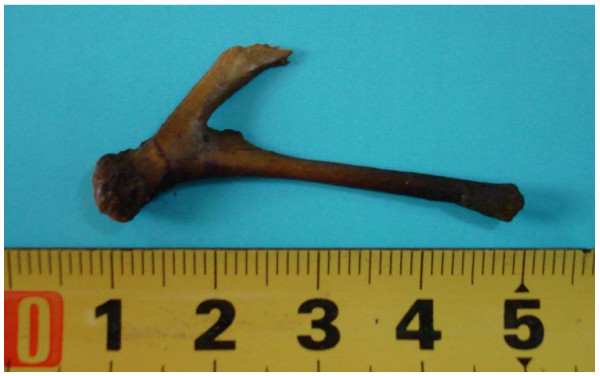
**Extracted chicken wishbone from ileum**.

During laparotomy we found diffuse fibro purulent peritonitis and adhesions. A tiny sharp-pointed object was found penetrating the inflamed portion of the distal ileum (Figure 2). Soon thereafter, a chicken wishbone was removed. We decided to do resection of the distal ileum and ileostomy, and four months later in the second operation we performed intestinal reconstruction. The postoperative treatment went well.

**Figure 3:**
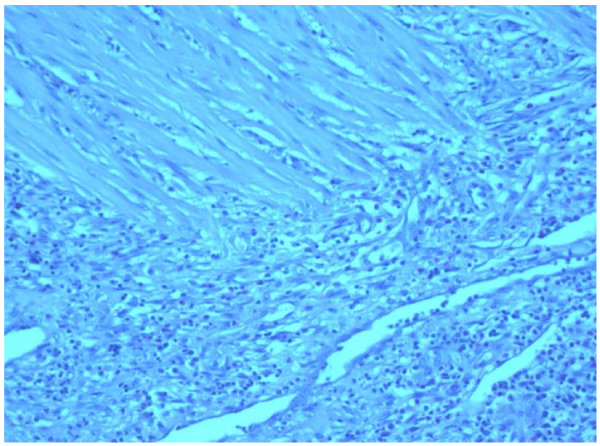
**Disrupted smooth muscles replaced by granulation tissue.** H&E stained, ×10.

The HP diagnosis revealed that a macroscopic and histological feature of examined samples responds to Ileitis non specific, or the perforation of the small intestine (Figure 3).

## Conclusion

Intestinal perforation by a chicken bone is rare and affects the left colon or distal ileum. The lack of information of ingestion and detection of chicken bones preoperatively are of interest to be considered in the differential diagnosis of acute abdomen, which in this case was treated surgically.

## Consent

We have written consent form from the patient for publication of this case report and accompanying images.

## Competing interests

The authors declare that they have no competing interests.

## Authors' contributions

FTH, SHIH, DSK and SHMH performed the surgery and general anesthesia. HLGL and FK has made Histopathology. FTH and ASK made substantial contributions to the concept, design and definition of intellectual content along with the literature search of the manuscript. All authors have participated sufficiently in the work to take public responsibility for appropriate portions of the content.

All authors read and approved the final manuscript.
